# Comparative Short-Term Clinical Events Associated with Molnupiravir and Ensitrelvir for COVID-19: A Retrospective Cohort Study Using Community Pharmacy Records in Japan

**DOI:** 10.3390/pharmacy14070148

**Published:** 2026-10-06

**Authors:** Kasumi Takabatake, Nobuhiro Ooba, Rie Nakajima, Masao Takahashi, Eiji Kawakami, Michiyo Kawana, Daiki Watanabe, Hajime Hashiba

**Affiliations:** 1 Department of Pharmacoepidemiology, School of Pharmacy, Nihon University, Funabashi, Chiba 274-8555, Japan; 2 Department of Pharmacy Practice in Primary Care, School of Pharmacy, Nihon University, Funabashi, Chiba 274-8555, Japan; 3 Japan Pharmaceutical Association, Shinjuku-ku, Tokyo 160-8389, Japan; 4 Department of Pharmaceutical Sciences, School of Pharmacy, Teikyo University, Itabashi-ku, Tokyo 173-8605, Japan

**Keywords:** COVID-19, SARS-CoV-2, molnupiravir, ensitrelvir, safety, real-world data, retrospective cohort study, drug event monitoring, community pharmacist

## Abstract

Molnupiravir and ensitrelvir are widely used for outpatient coronavirus disease 2019 treatment; however, real-world safety comparisons remain limited. This retrospective cohort study evaluated acute clinical event risks over a 29-day follow-up using community pharmacy records from Japanese patients newly prescribed either drug between January and September 2023. Hazard ratios (HRs) were estimated using multivariable Cox proportional hazard models. The analysis included 2051 patients prescribed molnupiravir and 563 prescribed ensitrelvir. Patients prescribed molnupiravir were older and had a higher prevalence of comorbidities than those prescribed ensitrelvir. However, after multivariable adjustment, no significant differences in the risk of major symptoms, such as diarrhea or vertigo, or overall add-on drug prescription were observed between the patient groups. An exception was anti-inflammatory drugs, with a significantly higher risk in the molnupiravir group (adjusted HR: 3.44, 95% confidence interval: 1.07–11.06). These results indicate that while short-term acute clinical event profiles were broadly comparable between molnupiravir and ensitrelvir in routine practice, the differences in add-on prescriptions, combined with the observational study design and limited statistical power in sub-analyses, highlight persistent clinical uncertainty. Our findings provide real-world evidence complementary to that from randomized controlled trials and emphasize the need for larger future studies.

## 1. Introduction

Coronavirus disease 2019 (COVID-19) is an infectious disease caused by severe acute respiratory syndrome coronavirus 2 (SARS-CoV-2) [1]. Following the World Health Organization’s declaration of a pandemic in early 2020, the rapid global spread of COVID-19 generated public anxiety owing to difficulties in controlling asymptomatic transmission [1,2]. Subsequent infection control measures, including nationwide lockdowns implemented in many countries, further exacerbated economic and social stress [3], while surges in severe cases placed unprecedented strain on healthcare systems [1,4]. Consequently, the pandemic has had a profound impact on global public health and socioeconomic stability [2,3,4]. Clinically, COVID-19 not only causes pneumonia with acute respiratory failure, but also systemic microvascular complications and persistent declines in quality of life associated with post-COVID-19 condition (long COVID) [3,5,6]. Therefore, timely and appropriate pharmacotherapy during the early stages of infection is crucial to improve patient outcomes [6,7].

Several therapeutic agents targeting SARS-CoV-2 infection have been developed, approved, and introduced into clinical practice. Remdesivir, an RNA-dependent polymerase inhibitor, was approved in several countries, including Japan, following Emergency Use Authorization in the United States in May 2020. Although it became a cornerstone of treatment for hospitalized patients, its role in early outpatient management of COVID-19 was limited because of the need for intravenous administration [8,9]. Subsequently, molnupiravir, an oral RNA-dependent polymerase inhibitor, was approved in the United Kingdom in November 2021 [10] and later introduced in the United States [11] and Japan [12] as outpatient treatment for patients with mild-to-moderate disease. In contrast, ensitrelvir, a selective 3CL protease inhibitor, received emergency regulatory approval in Japan [12,13,14,15] in November 2022, several years before its introduction in the United States in 2026. Although the use of these agents is guided by patient-specific risk factors for progression to severe disease [16], both have become important treatment options for the outpatient management of mild-to-moderate COVID-19.

In phase 3 trials comparing molnupiravir and placebo [17,18], the most commonly reported adverse events included gastrointestinal symptoms such as diarrhea, nausea, and vomiting, as well as dizziness and headache. In contrast, phase 2/3 and phase 3 randomized controlled trials comparing ensitrelvir and placebo [13,15] primarily reported abnormal laboratory values, including decreased high-density lipoprotein cholesterol and increased blood triglyceride levels, together with diarrhea and nausea. However, the extent to which the safety profiles of these two drugs differ remains unclear.

Although both drugs are widely used to treat SARS-CoV-2 infection in routine clinical practice in Japan, direct comparative data on their safety are limited. In this context, event monitoring by community pharmacists through regular patient follow-up to support medication adherence and provide appropriate medication counseling may facilitate the detection of adverse events, including symptoms that are often underrecognized or underreported [18].

Comparative studies of drugs with the same clinical indications are essential for appropriately evaluating the safety of medicines. Therefore, this retrospective cohort study aimed to evaluate the associations between prescribed treatment with either molnupiravir or ensitrelvir and subsequent recorded outcomes—including acute clinical events, symptom exacerbations, and add-on drug prescriptions—in real-world clinical practice in Japan. This study used prescription records from community pharmacies and pharmacist-collected patient records documenting treatment effectiveness and clinical events.

## 2. Materials and Methods

### 2.1. Study Design and Data Sources

This retrospective cohort study was conducted in February 2024 at 50,800 community pharmacies affiliated with the Japan Pharmaceutical Association. For the 4545 participating pharmacies, all patients prescribed the study drugs (molnupiravir or ensitrelvir) between 1 January and 30 September 2023 were identified, including those at 1071 pharmacies prescribing molnupiravir and 389 prescribing ensitrelvir. Eligible patients were operationally defined as new users at the index dispensing date if initiating a new acute 5-day course of either molnupiravir or ensitrelvir for COVID-19, with no concurrent or overlapping prescriptions of other oral antivirals. Individuals who did not revisit the pharmacy during the 29-day follow-up period were excluded from the primary cohort analysis (1144/3195 [35.8%] in the molnupiravir group and 902/1465 [61.6%] in the ensitrelvir group). To maximize the cohort size, we also included a small number of eligible patients who initiated the study drugs shortly after 30 September 2023, provided they met all inclusion criteria. All molnupiravir and ensitrelvir prescriptions were issued by attending licensed physicians at outpatient medical institutions (such as clinics or hospitals) following a clinical or laboratory diagnosis of COVID-19, in accordance with national Japanese treatment guidelines. Community pharmacists were responsible for dispensing the prescribed medications, providing patient counseling, and systematically collecting event data during follow-up.

Molnupiravir and ensitrelvir were sequentially introduced at designated medical institutions on 25 December 2021, and 23 November 2022, respectively [12]. Because both drugs are exclusively indicated for short-term (5-day) treatment of acute SARS-CoV-2 infection and their distribution was limited during the early launch period, all identified patients were considered new users at the index date [19]. To ensure appropriate assessment of treatment-related events, patients who did not revisit the pharmacy after the initial dispensing were excluded. Although new-user cohort designs typically apply a formal washout period to exclude prevalent users, a pre-defined washout period was not strictly required in this study because repeat or long-term use of these acute antiviral therapies within a short period is extremely unlikely in real-world clinical practice. Instead, a 6-month baseline lookback period preceding the initial dispensing date (index date) was used to capture baseline clinical characteristics, including age, sex, smoking status, alcohol consumption, over-the-counter (OTC) medication use, comorbidities, concomitant medications, SARS-CoV-2 vaccination history, and prior COVID-19-related hospitalizations or treatments.

Consistent with previous studies [20,21,22], information on events occurring after treatment initiation was also collected. To protect patient privacy, pharmacists anonymized all baseline and follow-up data by assigning study identification numbers before submitting the data via a web-based system.

### 2.2. Definition of Events

Using a methodology similar to that used in previous Japanese studies [20,21,22] on drug event monitoring, an event was defined as any occurrence, including clinical events, confirmed during the follow-up period. Events included clinical symptoms suspected to be adverse reactions to the study drugs, unexpected worsening of comorbidities, discontinuation or switching of treatment drugs, and addition of new drugs. Clinical information regarding drug discontinuation and add-on prescriptions was used to complement the event data.

Data analyzed in this study were retrospectively extracted from pre-existing community pharmacy records in February 2024, which were contemporaneously documented by pharmacists during or immediately following the treatment period using standardized drug event monitoring (DEM) questionnaires. Importantly, the protocol explicitly required pharmacists to confirm and document the complete absence of clinical events (“no events”) as essential data, thereby minimizing selective non-reporting. Events were identified and assessed based on standardized clinical criteria. To avoid outcome misclassification while retaining clinical relevance, events were excluded only if conclusively identified as entirely attributable to an unrelated external condition [23]. All reported events with at least one possible causal relationship—where attribution to either the study drug or underlying COVID-19 progression could not be ruled out—were included in the primary analysis.

Pharmacists used the Medical Dictionary for Regulatory Activities (MedDRA) version 27.0 to encode the event information described in the questionnaires [24]. Based on the reported descriptions, the MedDRA lowest-level terms were selected, converted to broad preferred terms, and counted as the incidence of events. In addition, pharmacists utilized records such as electronic medication histories to report data regarding the incidence dates of these events. Events coded under MedDRA terms related to drug efficacy (e.g., “Reduced therapeutic effect”) reflected patient-reported subjective perceptions during follow-up counseling (such as perceived insufficient symptom relief) or clinical observations by pharmacists, rather than protocol-defined objective efficacy endpoints such as viral clearance rates or standardized symptom severity scores.

### 2.3. Statistical Analysis

To describe patient characteristics, categorical variables are expressed as frequencies and percentages, whereas continuous variables are expressed as means and standard deviations (SDs). To evaluate baseline differences between the two groups, standardized mean differences (SMDs) were estimated for all covariates. An absolute SMD of > 0.1 was considered to indicate a meaningful difference between groups [25]. Given the limited number of outcome events and the modest sample size in the ensitrelvir group (n = 563), propensity score matching was not utilized to avoid substantial loss of statistical power.

A Cox proportional hazards model was used to estimate the hazard ratios (HRs) for the occurrence of events, with the ensitrelvir group as the reference. Both unadjusted and multivariable-adjusted HRs (aHRs) and 95% confidence intervals (CIs) were estimated for the molnupiravir group. All covariates with an absolute SMD > 0.1 were considered potential confounding factors and included in the multivariable models. Given the limited number of outcome events, covariate selection was guided by established risk factors and a stepwise approach to reduce the risk of model overfitting while accounting for potential confounding factors.

Although the administration period for both drugs was 5 days, the follow-up period for event evaluation was set to a maximum of 29 days from the initial dispensing date, consistent with previous studies evaluating COVID-19 treatments [17,26]. For each patient, the observation period began on the initial dispensing date and ended at the date of event occurrence, date of change or discontinuation of the study drug, date of the last visit, day 29 from the initial dispensing date, or the end of the observation period (31 December 2023), whichever occurred first.

As a sensitivity analysis, Cox regression analysis using Firth’s penalized likelihood method was performed for events with a small number of occurrences to ensure the stability of HR estimates (Tables S1 and S2). To account for potential confounding owing to substantial baseline imbalances resulting from real-world treatment, propensity score (PS) adjustment was performed. Individual PSs for receiving ensitrelvir versus molnupiravir were calculated using a multivariable logistic regression model incorporating 24 baseline covariates (demographic factors, comorbidities, co-medications, and SARS-CoV-2 vaccination history). Multivariable Cox proportional hazards regression models were fitted for each outcome, adjusting for the calculated PS as a continuous covariate alongside treatment assignment. To prevent model instability resulting from low event counts—particularly in subgroup analyses and sparse individual event categories—Firth’s penalized likelihood Cox proportional hazards regression was applied for all multivariable and PS-adjusted analyses to ensure finite and robust HR estimates.

Additionally, three pre-specified sensitivity and subgroup analyses were conducted to evaluate the robustness of our primary findings:

First, to evaluate potential selection bias introduced by excluding patients who did not revisit the pharmacy after initial dispensing, an extreme-bound sensitivity analysis was performed in the total initial population (*N* = 3195 for molnupiravir and *N* = 1465 for ensitrelvir). The range of potential event proportions was calculated under two boundary assumptions: a conservative scenario assuming zero events among all non-revisiting patients (0% assumption) and a worst-case scenario assuming that all non-revisiting patients experienced the event (100% assumption; Table S3).

Second, to systematically evaluate the impact of progressive adjustment, treatment effects were compared across unadjusted, age- and sex-adjusted, and PS-adjusted Cox proportional hazards models (Table S4).

Third, to address potential model extrapolation resulting from baseline age imbalance, a subgroup analysis restricted to patients aged ≥65 years was conducted using multivariable Cox proportional hazards regression with Firth’s penalty (Table S5).

Statistical significance was defined as *p* < 0.05. All statistical analyses were performed using SAS version 9.4 (SAS Institute Inc., Cary, NC, USA).

## 3. Results

### 3.1. Patient Characteristics

Among the 4660 patients initially identified (3195 in the molnupiravir group and 1465 in the ensitrelvir group), 2046 patients (43.9%) who visited the pharmacy only once on the initial prescription date and did not revisit were excluded. Specifically, 1144 patients (35.8%) in the molnupiravir group and 902 patients (61.6%) in the ensitrelvir group were excluded from the primary analysis, leaving a final analytical cohort of 2614 patients. Of these, 2051 and 563 patients were newly prescribed molnupiravir or ensitrelvir respectively, between 1 January and 30 September 2023. Table 1 presents the baseline characteristics of new users in both groups.

The mean (SD) age of patients in the molnupiravir group (73.7 [14.3] years) was significantly higher than that in the ensitrelvir group (49.2 [20.6] years; SMD: 1.380). The molnupiravir group also had higher prevalence rates for all comorbidities, including hypertension, diabetes mellitus, and dyslipidemia, as well as higher usage rates of all evaluated concomitant medications, including antihypertensive, antidiabetic, and lipid-lowering drugs. SARS-CoV-2 vaccination was also more common in the molnupiravir group.

### 3.2. Incidence of Events and Comparison of Hazard Ratios

An acute clinical event was reported to occur in 332 patients (16.2%, 95% CI: 15.0–17.8%) in the molnupiravir group and 84 patients (14.9%, 95% CI: 12.2–18.1%) in the ensitrelvir group. The number of events, incidence proportions, and HRs of the main reported events are presented in Table 2. After multivariable adjustment, no significant between-group differences in the risk of any evaluated event were observed. Cough was the most frequently reported acute clinical event in both groups, occurring in 74 patients (3.6%) in the molnupiravir group and 28 patients (5.0%) in the ensitrelvir group. With the ensitrelvir group as the reference, the multivariable aHR was 0.97 (95% CI: 0.56–1.68), indicating no significant difference between the two groups. A reduced therapeutic effect was reported in 22 patients (1.1%) in the molnupiravir group and 5 patients (0.9%) in the ensitrelvir group, with a multivariable aHR of 1.76 (95% CI: 0.54–5.80), indicating no significant difference. Furthermore, no significant differences were observed in the risks of gastrointestinal events, including diarrhea, nausea, and constipation, or neurological events such as vertigo.

An add-on prescription was reported to occur in 552 patients (26.9%, 95% CI: 25.0–28.9%) in the molnupiravir group and 156 patients (27.7%, 95% CI: 24.2–31.5%) in the ensitrelvir group. Table 3 presents the number of events, incidence proportions, and HRs of the main reported add-on drugs. The multivariable aHR for add-on prescription was 3.44 (95% CI: 1.07–11.06) in the molnupiravir group, indicating a significantly increased risk compared with that in the ensitrelvir group; however, no other significant differences in risk were observed between the two groups for all other add-on drugs after multivariable adjustment.

### 3.3. Sensitivity and Subgroup Analyses

As a sensitivity analysis, Cox regression analysis using Firth’s penalized likelihood method was performed for all events and add-on drugs. Consequently, the significant difference observed in the primary analysis for the addition of anti-inflammatory drugs was no longer evident, whereas the results for the other events and add-on drugs remained consistent with those of the primary analysis (Tables S1 and S2).

In the extreme-bound sensitivity analysis evaluated in the total initial population (including non-revisiting patients; *N* = 3195 for molnupiravir and *N* = 1465 for ensitrelvir), the estimated ranges of event proportions spanning from the conservative scenario (0% assumption) to the worst-case scenario (100% assumption) were 10.4% to 46.2% for molnupiravir and 5.7% to 67.3% for ensitrelvir for overall clinical events, and 17.3% to 53.1% and 10.6% to 72.2% for add-on prescriptions, respectively (Table S3). The true population event proportions strictly lay within these boundary limits, demonstrating that the observed comparative patterns must lie between the two drugs remained robust regardless of missing follow-up data.

In the propensity score-adjusted analysis incorporating 24 baseline covariates with Firth’s penalized Cox regression (Table S4), the PS-adjusted HRs for overall acute clinical events and add-on prescriptions were consistent with those of the primary multivariable Cox regression models. Unadjusted, age- and sex-adjusted, and PS-adjusted HRs showed similar directional trends. Notably, significantly higher risks of add-on prescriptions in the molnupiravir group were consistently observed for anti-inflammatory drugs (PS-adjusted HR: 3.64, 95% CI: 1.08–12.30) and anti-influenza drugs (PS-adjusted HR: 10.54, 95% CI: 1.10–100.87; Table S4).

In the subgroup analysis restricted to patients aged ≥65 years (Table S5), multivariable Cox regression with Firth’s penalty yielded consistent HR estimates. No unexpected safety signals or marked divergent trends were observed in older adults compared with the overall study population.

## 4. Discussion

This is the first retrospective cohort study to directly compare the risk of event occurrence between patients newly prescribed molnupiravir (*N* = 2051) or ensitrelvir (*N* = 563) to treat SARS-CoV-2 infection in real-world clinical practice in Japan, using primary data actively collected by community pharmacists. After multivariable adjustment for confounding factors, no significant differences were observed between the two groups for the risk of any evaluated event, including gastrointestinal symptoms, such as diarrhea, nausea, and constipation, and neurological events, such as vertigo, which is of particular concern during molnupiravir use. While these findings suggest that the safety profiles of the two oral antiviral drugs with respect to specific clinical symptoms may be similar in real-world clinical settings, the inherent baseline imbalances and potential preferential prescribing practices in the treatment groups warrant cautious interpretation of these comparative results.

The baseline characteristics differed substantially between the two groups, with SMDs exceeding 0.1 for most variables related to age, comorbidities, and concomitant medications, indicating substantial baseline imbalances. These differences are strongly rooted in the distinct regulatory indications, clinical contraindications, and historical distribution logistics of these drugs in Japan [16]. Molnupiravir received special approval for emergency use in December 2021 [12], with its indication restricted to patients aged ≥ 18 years with at least one risk factor for severe disease. In contrast, ensitrelvir, approved under the emergency regulatory framework in November 2022 [12], was indicated for patients aged ≥12 years regardless of risk factors. Furthermore, clinical contraindications and drug–drug interactions likely played a crucial role in these prescribing decisions. Molnupiravir is strictly contraindicated in pregnant women [27], whereas ensitrelvir has a wide array of contraindicated concomitant medications due to its strong drug interaction profile [28]. Consequently, physicians may have preferentially prescribed molnupiravir to older patients who, despite taking multiple medications for chronic comorbidities, had no pregnancy concerns, while selectively prescribing ensitrelvir to younger patients who were highly likely to be free of chronic underlying diseases, and thus had fewer concomitant prescriptions and lower risk of drug interactions.

In addition, the historical shift in the logistical and distribution landscape in Japan contributed to these prescribing patterns. Immediately following its approval, molnupiravir was managed under strict government allocation, and its distribution was limited to a very small number of registered community pharmacies, severely restricting its initial local accessibility. By the time ensitrelvir was launched, however, the distribution system had transitioned to general distribution channels, making both oral antivirals widely accessible across almost all community pharmacies. Although home delivery and doorstep placement of medications by pharmacists remained active throughout the pandemic to accommodate patient self-isolation, the crucial transition was the evolution from a highly restricted, registry-based pharmacy network to a broad, general community pharmacy distribution system that dramatically improved patient access. This transition in both the clinical decision-making and logistical frameworks explains the preferential prescribing practices (treatment-channeling) observed in our study: Molnupiravir was selectively prescribed to older, high-risk patients, whereas ensitrelvir was more broadly administered to a younger population with fewer or no chronic comorbidities. Given this substantial confounding inherent in real-world treatment selection, our finding of the lack of significant differences in event risk even after rigorous multivariable adjustments provides robust evidence of the clinical comparability between molnupiravir and ensitrelvir under proper statistical control.

Regarding add-on drug prescriptions, a significant difference between the two treatment groups was observed for anti-inflammatory drugs only, which exhibited a higher risk of prescription in the molnupiravir group (adjusted HR: 3.44, 95% CI: 1.07–11.06). Although this association did not reach significance in secondary sensitivity analyses, this non-significant result should be interpreted with caution. The small event numbers in the sensitivity analyses substantially reduced statistical power, increasing the risk of Type II errors rather than confirming a true absence of difference. Therefore, while overall add-on drug requirements appeared generally similar, our study was underpowered to draw definitive conclusions regarding specific drug classes, highlighting the need for larger studies to evaluate add-on prescription patterns.

The major strength of this study is the use of primary data that were retrospectively collected by community pharmacists using a standardized framework consistent with those in previous DEM studies [20,21,22]. This approach enabled the comprehensive capture of mild events that did not require hospitalization, such as diarrhea, nausea, and oral or pharyngeal pain, which may be underreported in spontaneous reporting systems [18] and are often not coded in large-scale claims databases. Furthermore, detailed clinical information collected during pharmacy-based medication counseling, including lifestyle factors (smoking status and alcohol consumption), use of OTC drugs, and SARS-CoV-2 vaccination history, was incorporated into the multivariable analysis. This facilitated a more comprehensive adjustment for potential confounding than would by typically possible using claims data alone.

While international large-scale clinical trials and real-world studies have evaluated individual antivirals—such as the global MOVe-OUT trial [17] and the UK PANORAMIC trial [26] for molnupiravir, or the Asian SCORPIO-SR trial [15] for ensitrelvir—direct comparative real-world evidence between molnupiravir and ensitrelvir remains extremely scarce. This scarcity is largely because ensitrelvir received emergency approval primarily in Japan ahead of broader global regulatory entries. Furthermore, while major international observational studies have predominantly focused on 28- to 30-day risks of hospitalization and mortality, our study uniquely utilized community pharmacy DEM with a 29-day follow-up period tailored specifically to capture short-term, acute clinical symptoms and medication add-ons in routine outpatient care. This Japanese pharmacy-based real-world dataset fills an international evidence gap regarding the relative acute safety of these two oral antivirals.

However, this study has certain limitations. First, because data were collected through patient interviews and medication histories at community pharmacies, without access to clinical laboratory results, objective assessment of blood test data reported in clinical trials of ensitrelvir [13,15], such as decreased high-density lipoprotein cholesterol and elevated blood triglycerides, was not possible. Second, although we adjusted for multiple confounding factors, including detailed patient lifestyle habits, OTC drug use, and vaccination status, the potential for residual confounding due to unmeasured clinical factors cannot be entirely ruled out. While we performed extensive multivariable adjustment and Firth’s sensitivity analysis to address this, unmeasured confounding factors such as the exact number of days from COVID-19 onset to treatment initiation and differences in circulating viral variants could not be accounted for. Third, consistent with previous studies, the events were assessed only during the 29-day period after dispensing and were determined by pharmacists during medication counseling. Therefore, this study could not adequately evaluate the effects of the two drugs on longer-term post-COVID-19 outcomes (long COVID) that may have spanned several months or more [6], as well as their effects on long-term prognosis. Fourth, analyses of add-on prescriptions (e.g., antipyretics and anti-inflammatory drugs) were conducted without formal adjustment for multiple comparisons, and their results should be cautiously interpreted as exploratory secondary outcomes. While these add-on medications are clinically valuable as complementary indicators of persistent symptoms, they may also reflect physician prescribing habits, routine symptom management, or unrelated clinical conditions rather than direct drug-related safety events. Fifth, patients who did not revisit the pharmacy after initial dispensing (35.8% for molnupiravir and 61.6% for ensitrelvir) were excluded to ensure follow-up assessment, which may have introduced selection bias due to differential loss to follow-up. Patients who experienced mild or no clinical events and achieved full recovery inherently had no reason to return, potentially inflating the observed event proportions among the analyzed cohort. Conversely, patients who experienced severe disease progression requiring hospitalization might have been lost to follow-up at the pharmacy level, which could have led to an underestimation of severe outcomes. To address this, our extreme-bound sensitivity analysis in the total initial population established upper and lower boundary limits (Table S3), confirming that while absolute event proportions vary depending on follow-up assumptions, the relative comparative safety trends between the two antivirals remained robust. Sixth, as this was an open-label real-world study, both patients and pharmacists were aware of the prescribed treatment, which may introduce observer or reporting bias. However, because event identification and recording were performed uniformly under a standardized pharmacy protocol, any resulting misclassification was likely non-differential between the two drug groups, which typically biases results toward the null rather than exaggerating comparative differences. Seventh, regarding the definition of new drug users, although a formal long-term lookback window was constrained by pharmacy record availability, only a small and balanced proportion of patients had recorded COVID-19 treatment in the preceding 6 months (4.1% in the ensitrelvir group vs. 5.7% in the molnupiravir group). Given that oral antivirals are prescribed as a short 5-day course for acute infection rather than chronic therapy, each index prescription is highly likely to represent an incident treatment course for a distinct acute episode, thereby minimizing prevalent user bias. Eighth, owing to substantial baseline imbalances (e.g., age SMD: 1.380) resulting from real-world prescribing trends in Japan, our multivariable Cox regression models inherently relied on statistical extrapolation across population subgroups with limited covariate overlap. Owing to the absence of severe drug–drug interactions and contraindications compared with other oral antivirals, molnupiravir is frequently prescribed to older patients with multiple comorbidities in daily clinical practice—a treatment channeling phenomenon also recognized in large-scale pragmatic trials [29]. Although propensity score adjustments, multivariable models, and age-restricted subgroup analyses (Tables S4 and S5) were rigorously applied to mitigate this confounding, this inherent constraint in baseline covariate overlap warrants cautious interpretation of the comparative HRs. Ninth, symptoms and clinical events were documented through pharmacist-conducted interviews and structured questionnaires during follow-up visits, rather than prospective daily patient symptom diaries. Although pharmacists recorded data contemporaneously during pharmaceutical care—including explicit confirmation of “no events”—this interview-based approach may still have been susceptible to recall bias regarding very mild or transient symptoms occurring between initial dispensing and follow-up. Consequently, the observed event proportions should be interpreted as estimates of clinically recognized and reported symptoms rather than as true incidence risk. Tenth, for rare individual events, standard multivariable regression with numerous covariates poses a risk of overfitting. Although we mitigated this risk by employing propensity score adjustment and Firth’s penalized likelihood methods, residual instability in effect estimates for extremely low-frequency events cannot be entirely ruled out. Eleventh, while patients were defined as new users at the index dispensing date for their current acute COVID-19 episode, pharmacy DEM records lack long-term longitudinal claims history (such as a 6-month or 1-year lookback period). Consequently, remote prior exposure to oral antivirals during earlier, independent COVID-19 infections cannot be completely ruled out, although overlapping or repeat prescriptions within the same acute episode were prevented by national prescribing guidance [16]. Twelfth, clinical events and symptoms were collected retrospectively during follow-up pharmacist interviews rather than through a standardized prospective daily symptom diary. Consequently, this data collection method is susceptible to recall bias and reporting bias, as patients might fail to recall mild or transient symptoms, or selectively report expected adverse effects. While absolute event incidence rates should therefore be interpreted with caution owing to potential measurement error, identical standardized questionnaires were applied across both treatment groups, ensuring that any reporting bias was non-differential between antivirals. Thirteenth, for certain individual symptoms with very low event counts (e.g., diarrhea [n = 17] and constipation [n = 11]), standard multivariable regression models severely violated the events-per-variable (≥10) guideline, resulting in wide confidence intervals due to model overfitting. Although propensity score adjustment (collapsing confounders into a single continuous covariate) and Firth’s penalized likelihood were applied to minimize overfitting and numerical instability (Table S4), HR estimates for rare individual symptoms should be interpreted with caution.

Despite these limitations, our findings suggest no marked differences in immediate safety profiles, particularly with regard to major acute gastrointestinal or neurological events, between the molnupiravir and ensitrelvir groups within the adjusted patient cohorts in this Japanese real-world setting. However, given the inherent differences in patients’ baseline characteristics and drug utilization patterns, these results should be interpreted with caution. Continued active event monitoring and medication adherence support by community pharmacists may contribute to the appropriate use and ongoing safety evaluation of novel therapeutics across patients of different ages and baseline risks of severe disease.

In the hierarchical framework of clinical evidence, randomized controlled trials (RCTs) represent the gold standard for establishing causal efficacy owing to their high internal validity. Indeed, landmark RCTs established the efficacy of molnupiravir on clinical outcomes and medical resource utilization [30], and large-scale platform trials evaluated real-world antiviral dynamics [29]. However, RCTs are inherently constrained by highly selective eligibility criteria and strictly controlled settings, which often limit their external validity and generalizability to routine care. Conversely, while observational real-world studies may exhibit lower internal validity due to potential residual confounding, they offer superior external validity and generalizability by evaluating unselected, heterogeneous outpatient populations in daily practice. As a retrospective cohort study in clinical real-world settings utilizing community pharmacy DEM data, our study occupies a complementary position within the evidence hierarchy. Rather than offering definitive causal proof, it provides valuable real-world evidence that enhances generalizability and complements RCT findings to inform routine prescribing decisions.

## 5. Conclusions

Our pharmacy-based real-world study demonstrates broadly comparable short-term acute clinical event profiles between molnupiravir and ensitrelvir in routine outpatient COVID-19 care. However, given the observational study design, potential residual confounding, and limited statistical power in sub-analyses due to small event counts, these findings do not establish definitive causal equivalence or superiority. Rather, as real-world evidence that complements findings from RCTs, these results provide valuable insights into daily clinical practice while carrying inherent clinical and statistical uncertainty. Therefore, our comparative findings should be interpreted with caution, highlighting the necessity for larger, multi-center real-world investigations to further clarify outpatient tolerability and prescribing patterns.

## Figures and Tables

**Table 1:** Baseline characteristics of new users of molnupiravir or ensitrelvir.

	Molnupiravir (*N* = 2051)	Ensitrelvir (*N* = 563)	Standardized Mean Difference (SMD)
Age (years), mean (SD)	73.7 (14.3)	49.2 (20.6)	1.380
Male sex, n (%)	905 (44.1)	242 (43.0)	0.023
Lifestyle factor, n (%)			
Smoking	103 (5.0)	37 (6.6)	0.066
Alcohol consumption	390 (19.0)	121 (21.5)	0.062
OTC drug use	36 (1.8)	16 (2.8)	0.073
Comorbidities, n (%)			
Hypertension	1184 (57.7)	122 (21.7)	0.793
Diabetes mellitus	388 (18.9)	38 (6.7)	0.370
Dyslipidemia	737 (35.9)	79 (14.0)	0.523
Hyperuricemia	206 (10.0)	21 (3.7)	0.251
Myocardial infarction	55 (2.7)	4 (0.7)	0.153
Heart failure	128 (6.2)	9 (1.6)	0.241
Peptic ulcer	235 (11.5)	23 (4.1)	0.278
Asthma	84 (4.1)	19 (3.4)	0.038
Cancer	77 (3.8)	5 (0.9)	0.191
Chronic kidney disease	113 (5.5)	3 (0.5)	0.294
Liver diseases	47 (2.3)	2 (0.4)	0.170
Medical history, n (%)			
Hospital admission (any cause)	17 (0.8)	1 (0.2)	0.092
SARS-CoV-2 vaccination	722 (35.2)	117 (20.8)	0.325
COVID-19 treatment	116 (5.7)	23 (4.1)	0.073
COVID-19 hospital admission	1 (0.0)	0 (0)	0.031
Co-medications, n (%)			
Antihypertensives	1200 (58.5)	121 (21.5)	0.816
Antidiabetics, except insulin	371 (18.1)	36 (6.4)	0.363
Insulin	53 (2.6)	4 (0.7)	0.148
Lipid-lowering agents	741 (36.1)	78 (13.9)	0.532
Hypouricemic agents	219 (10.7)	22 (3.9)	0.263
Antipsychotics	178 (8.7)	18 (3.2)	0.234
Hypnotics	279 (13.6)	27 (4.8)	0.308
Antiulceratives	459 (22.4)	37 (6.6)	0.461
Steroids except those for external use	49 (2.4)	3 (0.5)	0.155

Abbreviations: COVID-19, Coronavirus disease 2019; OTC, over-the-counter; SARS-CoV-2, severe acute respiratory syndrome coronavirus 2; SD, standard deviation.

**Table 2:** Number, incidence proportions, and hazard ratios of main events reported by new users of molnupiravir or ensitrelvir.

Events			Hazard Ratio (95% Confidence Interval)		
	Molnupiravir (*N* = 2051) *n* (%)	Ensitrelvir (*N* = 563) *n* (%)	Unadjusted	Age and Sex Adjusted	Multivariable Adjusted ^a^
Cough	74 (3.6)	28 (5.0)	0.68 (0.44–1.05)	0.87 (0.51–1.48)	0.97 (0.56–1.68)
Reduced therapeutic effect	22 (1.1)	5 (0.9)	1.12 (0.42–2.95)	1.58 (0.50–5.01)	1.76 (0.54–5.80)
Productive cough	19 (0.9)	11 (2.0)	0.44 (0.21–0.93)	0.71 (0.28–1.81)	0.76 (0.29–2.00)
Oral and pharyngeal pain	14 (0.7)	13 (2.3)	0.28 (0.13–0.58)	0.35 (0.14–0.89)	0.52 (0.20–1.38)
Nasopharyngitis	14 (0.7)	4 (0.7)	0.87 (0.29–2.64)	1.86 (0.47–7.28)	2.10 (0.50–8.77)
Diarrhea	13 (0.6)	4 (0.7)	0.86 (0.28–2.62)	1.94 (0.49–7.73)	2.34 (0.57–9.65)
Vertigo	13 (0.6)	2 (0.4)	1.63 (0.37–7.21)	2.23 (0.40–12.41)	2.29 (0.40–13.08)
Elevated blood pressure	10 (0.5)	1 (0.2)	2.47 (0.32–19.27)	2.03 (0.22–18.96)	1.45 (0.16–12.95)
Fever	10 (0.5)	5 (0.9)	0.50 (0.17–1.47)	0.30 (0.09–0.99)	0.32 (0.09–1.11)
Constipation	10 (0.5)	1 (0.2)	2.50 (0.32–19.52)	1.53 (0.17–13.70)	1.67 (0.18–15.80)
Nausea	9 (0.4)	4 (0.7)	0.59 (0.18–1.90)	0.41 (0.11–1.62)	0.52 (0.13–2.14)
Rash	9 (0.4)	2 (0.4)	1.19 (0.26–5.50)	1.73 (0.28–10.62)	1.80 (0.28–11.37)
Phlegm accumulation	8 (0.4)	1 (0.2)	2.03 (0.25–16.23)	0.86 (0.10–7.58)	0.69 (0.08–6.14)
Itching	7 (0.3)	1 (0.2)	1.84 (0.23–14.92)	2.10 (0.19–22.76)	2.19 (0.19–24.71)
Fatigue	7 (0.3)	3 (0.5)	0.59 (0.15–2.28)	0.33 (0.07–1.51)	0.37 (0.08–1.80)
Influenza	5 (0.2)	3 (0.5)	0.41 (0.10–1.72)	3.19 (0.53–19.11)	4.28 (0.68–26.88)
Upper abdominal pain	5 (0.2)	2 (0.4)	0.64 (0.12–3.28)	1.16 (0.15–9.07)	1.34 (0.16–11.56)
Rhinitis	5 (0.2)	4 (0.7)	0.32 (0.09–1.18)	0.43 (0.08–2.15)	0.45 (0.08–2.40)
Abdominal discomfort	4 (0.2)	1 (0.2)	1.02 (0.11–9.11)	2.03 (0.14–29.38)	1.55 (0.11–22.80)
Shortness of breath	3 (0.1)	1 (0.2)	0.75 (0.08–7.20)	0.54 (0.04–7.27)	0.70 (0.03–13.76)
Headache	3 (0.1)	4 (0.7)	0.19 (0.04–0.86)	0.34 (0.05–2.20)	0.28 (0.04–1.83)
Anemia	3 (0.1)	2 (0.4)	0.37 (0.06–2.22)	0.57 (0.06–5.36)	0.38 (0.04–3.65)

^a^ Adjusted for age, sex, alcohol consumption, over-the-counter drug use, hypertension, diabetes mellitus, dyslipidemia, myocardial infarction, heart failure, peptic ulcer, asthma, cancer, hyperuricemia, chronic kidney disease, liver disease, history of hospital admission, concomitant medications (antihypertensives, antidiabetics, lipid-lowering agents, antipsychotics, hypnotics, antiulceratives, and steroids except those for external use), and severe acute respiratory syndrome coronavirus 2 vaccination.

**Table 3:** Number, incidence proportions, and hazard ratios of add-on prescriptions for new users of molnupiravir or ensitrelvir.

Add-On Prescriptions			Hazard Ratio (95% Confidence Interval)		
	Molnupiravir (*N* = 2051) *n* (%)	Ensitrelvir (*N* = 563) *n* (%)	Unadjusted	Age and Sex Adjusted	Multivariable Adjusted ^a^
Expectorants	62 (3.0)	21 (3.7)	0.72 (0.44–1.19)	0.88 (0.48–1.60)	0.96 (0.52–1.78)
Antipyretics and analgesics	38 (1.9)	22 (3.9)	0.41 (0.24–0.70)	0.56 (0.29–1.07)	0.67 (0.34–1.31)
Antitussives	36 (1.8)	16 (2.8)	0.56 (0.31–1.00)	1.20 (0.57–2.52)	1.26 (0.58–2.73)
Antiallergic agents	35 (1.7)	13 (2.3)	0.66 (0.35–1.25)	1.51 (0.68–3.36)	1.66 (0.72–3.79)
Antiulceratives	33 (1.6)	8 (1.4)	1.00 (0.46–2.16)	1.12 (0.45–2.78)	1.34 (0.52–3.42)
Antitussives and expectorants	26 (1.3)	5 (0.9)	1.29 (0.50–3.36)	1.96 (0.64–6.06)	2.33 (0.74–7.34)
Beta-lactam antibiotics	23 (1.1)	8 (1.4)	0.68 (0.31–1.53)	0.66 (0.26–1.72)	0.65 (0.24–1.73)
Antihypertensives	23 (1.1)	3 (0.5)	1.81 (0.54–6.04)	1.21 (0.33–4.48)	0.99 (0.27–3.64)
Anti-inflammatory drugs	15 (0.7)	6 (1.1)	0.63 (0.24–1.62)	2.50 (0.76–8.23)	3.44 (1.07–11.06)
Fluoroquinolones	13 (0.6)	5 (0.9)	0.63 (0.23–1.78)	0.49 (0.15–1.64)	0.46 (0.13–1.57)
Probiotics	13 (0.6)	2 (0.4)	1.59 (0.36–7.05)	1.39 (0.26–7.45)	1.88 (0.33–10.75)
Macrolides	11 (0.5)	2 (0.4)	1.31 (0.29–5.92)	1.68 (0.29–9.69)	1.61 (0.26–9.89)
Magnesium laxatives	9 (0.4)	1 (0.2)	2.20 (0.28–17.34)	2.09 (0.21–20.66)	2.24 (0.22–22.57)
Steroids	8 (0.4)	1 (0.2)	1.97 (0.25–15.76)	3.82 (0.36–40.97)	4.95 (0.44–56.02)
Antiemetics	7 (0.3)	4 (0.7)	0.43 (0.13–1.46)	0.31 (0.07–1.29)	0.35 (0.08–1.53)
Anti-influenza drugs	6 (0.3)	1 (0.2)	1.42 (0.17–11.75)	8.18 (0.74–89.84)	10.31 (0.91–117.49)
Antivertigo agents	6 (0.3)	2 (0.4)	0.70 (0.14–3.49)	0.94 (0.13–6.59)	1.14 (0.16–7.93)
Common cold drugs	3 (0.1)	2 (0.4)	0.38 (0.06–2.25)	2.71 (0.24–30.04)	1.81 (0.17–19.61)

^a^ Adjusted for age, sex, alcohol consumption, over-the-counter drug use, hypertension, diabetes mellitus, dyslipidemia, myocardial infarction, heart failure, peptic ulcer, asthma, cancer, hyperuricemia, chronic kidney disease, liver disease, history of hospital admission, concomitant medications (antihypertensives, antidiabetics, lipid-lowering agents, antipsychotics, hypnotics, antiulceratives, and steroids except those for external use), and severe acute respiratory syndrome coronavirus 2 vaccination.

## Data Availability

The data presented in this study are available on request from the corresponding author due to patient privacy and ethical restrictions by the Ethics Committee of the Japan Pharmaceutical Association.

## Supplementary Materials

The following supporting information can be downloaded at https://www.mdpi.com/article/10.3390/pharmacy14070148/s1, Table S1: Sensitivity analysis using Firth’s penalized likelihood approach for the incidence of acute clinical events that were common between molnupiravir and ensitrelvir users; Table S2: Sensitivity analysis for any add-on prescriptions using Firth’s penalized Cox regression model; Table S3: Sensitivity analysis for acute clinical events and add-on prescriptions in the study population (revisiting patients) versus the total initial population (including non-revisiting patients); Table S4: Unadjusted, age- and sex-adjusted, and propensity score-adjusted hazard ratios for clinical events and add-on prescriptions associated with molnupiravir versus ensitrelvir use; Table S5: Subgroup analysis in patients aged ≥ 65 years: Unadjusted, age- and sex-adjusted, and multivariable-adjusted hazard ratios for acute clinical events and add-on prescriptions associated with molnupiravir versus ensitrelvir use.

## Abbreviations

The following abbreviations are used in this manuscript:

aHR	adjusted HR
CI	confidence interval
COVID-19	Coronavirus disease 2019
DEM	drug event monitoring
HR	hazard ratio
MedDRA	Medical Dictionary for Regulatory Activities
OTC	over-the-counter
PS	propensity score
RCT	randomized controlled trial
SARS-CoV-2	severe acute respiratory syndrome coronavirus 2
SD	standard deviation
SMD	standardized mean difference
