# Supplementary material for: Comparative Short-Term Clinical Events Associated with Molnupiravir and Ensitrelvir for COVID-19: A Retrospective Cohort Study Using Community Pharmacy Records in Japan
Source: Pharmacy (Basel). 2026 Oct 6;14(7):148. doi: 10.3390/pharmacy14070148 (PMC13708640; doi:10.3390/pharmacy14070148)
Supplement: Supplementary file 1 [file pharmacy-14-00148-s001.zip › pharmacy-4563291-supplementary.pdf]

Supplementary Information

Table S1. Sensitivity analysis using Firth's penalized likelihood approach for the incidence of acute clinical events that were common between molnupiravir and ensitrelvir users.

| Acute clinical events | Molnupiravir (*N*= 2051) *n* (%) | Ensitrelvir (*N* = 563) *n* (%) | Hazard ratio (95% Confidence Interval) | | |
|---|---|---|---|---|---|
| | | | **Unadjusted** | **Age and sex adjusted** | **Multivariate adjusted** |
| Cough | 74 (3.6) | 28 (5.0) | 0.67 (0.44–1.04) | 0.86 (0.51–1.46) | 0.96 (0.55–1.67) |
| Reduced therapeutic effect | 22 (1.1) | 5 (0.9) | 1.04 (0.40–2.69) | 1.47 (0.48–4.54) | 1.67 (0.51–5.51) |
| Productive cough | 19 (0.9) | 11 (2.0) | 0.43 (0.21–0.91) | 0.70 (0.28–1.77) | 0.75 (0.29–1.97) |
| Oral and pharyngeal pain | 14 (0.7) | 13 (2.3) | 0.27 (0.13–0.58) | 0.35 (0.14–0.88) | 0.52 (0.20–1.36) |
| Nasopharyngitis | 14 (0.7) | 4 (0.7) | 0.80 (0.27–2.37) | 1.71 (0.45–6.56) | 1.95 (0.46–8.35) |
| Diarrhea | 13 (0.6) | 4 (0.7) | 0.79 (0.26–2.37) | 1.80 (0.46–7.00) | 2.17 (0.52–9.13) |
| Vertigo | 13 (0.6) | 2 (0.4) | 1.35 (0.34–5.45) | 1.85 (0.37–9.33) | 1.92 (0.35–10.48) |
| Elevated blood pressure | 10 (0.5) | 1 (0.2) | 1.72 (0.29–10.29) | 1.40 (0.20–9.88) | 1.04 (0.15–7.29) |
| Fever | 10 (0.5) | 5 (0.9) | 0.48 (0.17–1.40) | 0.28 (0.08–0.92) | 0.31 (0.09–1.06) |
| Constipation | 10 (0.5) | 1 (0.2) | 1.75 (0.29–10.42) | 1.05 (0.16–7.08) | 1.20 (0.16–8.96) |
| Nausea | 9 (0.4) | 4 (0.7) | 0.55 (0.17–1.76) | 0.38 (0.10–1.47) | 0.48 (0.12–1.98) |
| Rash | 9 (0.4) | 2 (0.4) | 1.00 (0.23–4.30) | 1.46 (0.26–8.32) | 1.53 (0.25–9.45) |
| Phlegm accumulation | 8 (0.4) | 1 (0.2) | 1.44 (0.23–8.96) | 0.60 (0.09–4.03) | 0.51 (0.08–3.54) |

| | | | | | |
|---|---|---|---|---|---|
| Itching | 7 (0.3) | 1 (0.2) | 1.31 (0.20–8.42) | 1.49 (0.17–12.67) | 1.69 (0.18–15.83) |
| Fatigue | 7 (0.3) | 3 (0.5) | 0.54 (0.14–2.05) | 0.30 (0.07–1.33) | 0.34 (0.07–1.69) |
| Influenza | 5 (0.2) | 3 (0.5) | 0.39 (0.09–1.61) | 3.02 (0.50–18.31) | 3.88 (0.50–29.83) |
| Upper abdominal pain | 5 (0.2) | 2 (0.4) | 0.56 (0.11–2.77) | 1.01 (0.14–7.50) | 1.17 (0.13–10.76) |
| Rhinitis | 5 (0.2) | 4 (0.7) | 0.31 (0.08–1.15) | 0.41 (0.08–2.04) | 0.42 (0.08–2.28) |
| Abdominal discomfort | 4 (0.2) | 1 (0.2) | 0.76 (0.10–5.78) | 1.50 (0.12–18.37) | 1.20 (0.08–17.48) |
| Shortness of breath | 3 (0.1) | 1 (0.2) | 0.58 (0.07–4.94) | 0.41 (0.04–4.72) | 0.52 (0.03–9.66) |
| Headache | 3 (0.1) | 4 (0.7) | 0.20 (0.05–0.89) | 0.33 (0.05–2.17) | 0.29 (0.05–1.84) |
| Anemia | 3 (0.1) | 2 (0.4) | 0.35 (0.06–2.05) | 0.51 (0.06–4.67) | 0.35 (0.04–3.26) |

[a]Adjusted for age, sex, alcohol consumption, over-the-counter drug use, hypertension, diabetes mellitus, dyslipidemia, myocardial infarction, heart failure, peptic ulcer, asthma, cancer, hyperuricemia, chronic kidney disease, liver disease, history of hospital admission, concomitant medications (antihypertensives, antidiabetics, lipid-lowering agents, antipsychotics, hypnotics, antiulceratives, and steroids except those for external use), and severe acute respiratory syndrome coronavirus 2 vaccination.

Table S2. Sensitivity analysis for any add-on prescriptions using Firth's penalized Cox regression model.

| **Add-on prescriptions** | **Molnupiravir (*N*= 2051) *n* (%)** | **Ensitrelvir (*N* = 563) *n* (%)** | **Hazard ratio (95% Confidence Interval)** | | |
|---|---|---|---|---|---|
| | | | **Unadjusted** | **Age and sex adjusted** | **Multivariate adjusted** |
| Expectorants | 62 (3.0) | 21 (3.7) | 0.71 (0.44–1.17) | 0.86 (0.48–1.56) | 0.94 (0.51–1.75) |
| Antipyretics and analgesics | 38 (1.9) | 22 (3.9) | 0.41 (0.24–0.69) | 0.55 (0.29–1.05) | 0.66 (0.33–1.30) |
| Antitussives | 36 (1.8) | 16 (2.8) | 0.55 (0.30–0.99) | 1.17 (0.56–2.47) | 1.24 (0.57–2.71) |
| Antiallergic agents | 35 (1.7) | 13 (2.3) | 0.65 (0.34–1.22) | 1.47 (0.66–3.26) | 1.63 (0.71–3.74) |
| Antiulceratives | 33 (1.6) | 8 (1.4) | 0.95 (0.44–2.04) | 1.06 (0.43–2.61) | 1.29 (0.50–3.29) |
| Antitussives and expectorants | 26 (1.3) | 5 (0.9) | 1.19 (0.47–3.04) | 1.82 (0.60–5.48) | 2.17 (0.69–6.86) |
| Beta-lactam antibiotics | 23 (1.1) | 8 (1.4) | 0.66 (0.30–1.46) | 0.63 (0.25–1.63) | 0.63 (0.24–1.67) |
| Antihypertensives | 23 (1.1) | 3 (0.5) | 1.58 (0.50–4.96) | 1.05 (0.30–3.64) | 0.87 (0.25–3.06) |
| Anti-inflammatory drugs | 15 (0.7) | 6 (1.1) | 0.60 (0.23–1.53) | 2.40 (0.73–7.84) | 3.30 (0.98–11.07) |
| Fluoroquinolones | 13 (0.6) | 5 (0.9) | 0.60 (0.22–1.66) | 0.46 (0.14–1.50) | 0.43 (0.13–1.48) |
| Probiotics | 13 (0.6) | 2 (0.4) | 1.32 (0.33–5.33) | 1.15 (0.24–5.56) | 1.57 (0.29–8.53) |
| Macrolides | 11 (0.5) | 2 (0.4) | 1.10 (0.27–4.53) | 1.39 (0.26–7.35) | 1.39 (0.23–8.30) |
| Magnesium laxatives | 9 (0.4) | 1 (0.2) | 1.54 (0.25–9.40) | 1.46 (0.19–11.08) | 1.59 (0.19–13.23) |
| Steroids | 8 (0.4) | 1 (0.2) | 1.40 (0.22–8.70) | 2.74 (0.32–23.27) | 3.55 (0.34–36.69) |

| | | | | | |
|---|---|---|---|---|---|
| Antiemetics | 7 (0.3) | 4 (0.7) | 0.41 (0.12–1.38) | 0.29 (0.07–1.20) | 0.33 (0.07–1.43) |
| Anti-influenza drugs | 6 (0.3) | 1 (0.2) | 1.02 (0.15–6.82) | 6.06 (0.65–56.17) | 7.81 (0.59–104.08) |
| Antivertigo agents | 6 (0.3) | 2 (0.4) | 0.61 (0.13–2.87) | 0.80 (0.12–5.27) | 0.94 (0.13–6.62) |
| Common cold drugs | 3 (0.1) | 2 (0.4) | 0.25 (0.06–2.08) | 2.50 (0.23–27.16) | 1.58 (0.12–20.13) |

[a]Adjusted for age, sex, alcohol consumption, over-the-counter drug use, hypertension, diabetes mellitus, dyslipidemia, myocardial infarction, heart failure, peptic ulcer, asthma, cancer, hyperuricemia, chronic kidney disease, liver disease, history of hospital admission, concomitant medications (antihypertensives, antidiabetics, lipid-lowering agents, antipsychotics, hypnotics, antiulceratives, and steroids except those for external use), and severe acute respiratory syndrome coronavirus 2 vaccination.

Table S3. Sensitivity analysis for acute clinical events and add-on prescriptions in the study population (revisiting patients) versus the total initial population (including non-revisiting patients).

| Events | **Study population (revisiting patients)** | | **Total initial population (including non-revisiting patients)** | |
|---|---|---|---|---|
| | **Molnupiravir (*n* = 2051) *n* (%)** | **Ensitrelvir (*n* = 563) *n* (%)** | **Molnupiravir (*N* = 3195), Estimated Range (%)** | **Ensitrelvir (*N* = 1465), Estimated Range (%)** |
| Acute clinical events | | | | |
| Any event | 332 (16.2) | 84 (14.9) | 10.4% to 46.2% | 5.7% to 67.3% |
| Cough | 74 (3.6) | 28 (5.0) | 2.3% to 38.1% | 1.9% to 63.5% |
| Reduced therapeutic effect | 22 (1.1) | 5 (0.9) | 0.7% to 36.5% | 0.3% to 61.9% |
| Productive cough | 19 (0.9) | 11 (2.0) | 0.6% to 36.4% | 0.8% to 62.3% |
| Oral and pharyngeal pain | 14 (0.7) | 13 (2.3) | 0.4% to 36.2% | 0.9% to 62.5% |
| Nasopharyngitis | 14 (0.7) | 4 (0.7) | 0.4% to 36.2% | 0.3% to 61.8% |
| Diarrhea | 13 (0.6) | 4 (0.7) | 0.4% to 36.2% | 0.3% to 61.8% |
| Vertigo | 13 (0.6) | 2 (0.4) | 0.4% to 36.2% | 0.1% to 61.7% |
| Elevated blood pressure | 10 (0.5) | 1 (0.2) | 0.3% to 36.1% | 0.1% to 61.6% |
| Fever | 10 (0.5) | 5 (0.9) | 0.3% to 36.1% | 0.3% to 61.9% |
| Constipation | 10 (0.5) | 1 (0.2) | 0.3% to 36.1% | 0.1% to 61.6% |
| Nausea | 9 (0.4) | 4 (0.7) | 0.3% to 36.1% | 0.3% to 61.8% |
| Rash | 9 (0.4) | 2 (0.4) | 0.3% to 36.1% | 0.1% to 61.7% |
| Phlegm accumulation | 8 (0.4) | 1 (0.2) | 0.3% to 36.1% | 0.1% to 61.6% |
| Itching | 7 (0.3) | 1 (0.2) | 0.2% to 36.0% | 0.1% to 61.6% |
| Fatigue | 7 (0.3) | 3 (0.5) | 0.2% to 36.0% | 0.2% to 61.8% |
| Influenza | 5 (0.2) | 3 (0.5) | 0.2% to 36.0% | 0.2% to 61.8% |
| Upper abdominal pain | 5 (0.2) | 2 (0.4) | 0.2% to 36.0% | 0.1% to 61.7% |
| Rhinitis | 5 (0.2) | 4 (0.7) | 0.2% to 36.0% | 0.3% to 61.8% |

| | | | | |
|---|---|---|---|---|
| Abdominal discomfort | 4 (0.2) | 1 (0.2) | 0.1% to 35.9% | 0.1% to 61.6% |
| Shortness of breath | 3 (0.1) | 1 (0.2) | 0.1% to 35.9% | 0.1% to 61.6% |
| Headache | 3 (0.1) | 4 (0.7) | 0.1% to 35.9% | 0.3% to 61.8% |
| Anemia | 3 (0.1) | 2 (0.4) | 0.1% to 35.9% | 0.1% to 61.7% |
| Add-on prescriptions | | | | |
| Any prescription | 552 (26.9) | 156 (27.7) | 17.3% to 53.1% | 10.6% to 72.2% |
| Expectorants | 62 (3.0) | 21 (3.7) | 1.9% to 37.7% | 1.4% to 63.0% |
| Antipyretics and analgesics | 38 (1.9) | 22 (3.9) | 1.2% to 37.0% | 1.5% to 63.1% |
| Antitussives | 36 (1.8) | 16 (2.8) | 1.1% to 36.9% | 1.1% to 62.7% |
| Antiallergic agents | 35 (1.7) | 13 (2.3) | 1.1% to 36.9% | 0.9% to 62.5% |
| Antiulceratives | 33 (1.6) | 8 (1.4) | 1.0% to 36.8% | 0.5% to 62.1% |
| Antitussives and expectorants | 26 (1.3) | 5 (0.9) | 0.8% to 36.6% | 0.3% to 61.9% |
| Beta-lactam antibiotics | 23 (1.1) | 8 (1.4) | 0.7% to36.5% | 0.5% to 62.1% |
| Antihypertensives | 23 (1.1) | 3 (0.5) | 0.7% to36.5% | 0.2% to 61.8% |
| Anti-inflammatory drugs | 15 (0.7) | 6 (1.1) | 0.5% to 36.3% | 0.4% to 62.0% |
| Fluoroquinolones | 13 (0.6) | 5 (0.9) | 0.4% to 36.2% | 0.3% to 61.9% |
| Probiotics | 13 (0.6) | 2 (0.4) | 0.4% to 36.2% | 0.1% to 61.7% |
| Macrolides | 11 (0.5) | 2 (0.4) | 0.3% to 36.2% | 0.1% to 61.7% |
| Magnesium laxatives | 9 (0.4) | 1 (0.2) | 0.3% to 36.1% | 0.1% to 61.6% |
| Steroids | 8 (0.4) | 1 (0.2) | 0.3% to 36.1% | 0.1% to 61.6% |
| Antiemetics | 7 (0.3) | 4 (0.7) | 0.2% to 36.0% | 0.3% to 61.8% |
| Anti-influenza drugs | 6 (0.3) | 1 (0.2) | 0.2% to 36.0% | 0.1% to 61.6% |
| Antivertigo agents | 6 (0.3) | 2 (0.4) | 0.2% to 36.0% | 0.1% to 61.7% |
| Common cold drugs | 3 (0.1) | 2 (0.4) | 0.1% to 35.9% | 0.1% to 61.7% |

Abbreviations: N, number of total initial patients; n, number of patients analyzed.

* Estimated Range (%) represents the extreme bounds of potential event proportions in the total

initial population (including non-revisiting patients). The lower bound assumes zero events among non-revisiting patients (conservative scenario: observed events / total N), whereas the upper bound assumes that all non-revisiting patients experienced the event (worst-case scenario: [observed events + non-revisiting N] / total N).

Table S4. Unadjusted, age- and sex-adjusted, and propensity score-adjusted hazard ratios for clinical events and add-on prescriptions associated with molnupiravir versus ensitrelvir use.

| **Events** | | | **Hazard ratio[a] (95% Confidence Interval)** | | |
|---|---|---|---|---|---|
| | **Molnupiravir (*N* = 2051) *n* (%)** | **Ensitrelvir (*N* = 563) *n* (%)** | **Unadjusted** | **Age and sex adjusted** | **PS[a] adjusted[b]** |
| Acute clinical events | | | | | |
| Cough | 74 (3.6) | 28 (5.0) | 0.67 (0.44–1.04) | 0.86 (0.51–1.46) | 1.06 (0.60–1.89) |
| Reduced therapeutic effect | 22 (1.1) | 5 (0.9) | 1.04 (0.40–2.69) | 1.47 (0.48–4.54) | 2.45 (0.72–8.40) |
| Productive cough | 19 (0.9) | 11 (2.0) | 0.43 (0.21–0.91) | 0.70 (0.28–1.77) | 0.85 (0.31–2.34) |
| Oral and pharyngeal pain | 14 (0.7) | 13 (2.3) | 0.27 (0.13–0.58) | 0.35 (0.14–0.88) | 0.42 (0.16–1.16) |
| Nasopharyngitis | 14 (0.7) | 4 (0.7) | 0.80 (0.27–2.37) | 1.71 (0.45–6.56) | 1.90 (0.44–8.14) |
| Diarrhea | 13 (0.6) | 4 (0.7) | 0.79 (0.26–2.37) | 1.80 (0.46–7.00) | 1.95 (0.45–8.36) |
| Vertigo | 13 (0.6) | 2 (0.4) | 1.35 (0.34–5.45) | 1.85 (0.37–9.33) | 1.53 (0.28–8.41) |
| Elevated blood pressure | 10 (0.5) | 1 (0.2) | 1.72 (0.29–10.29) | 1.40 (0.20–9.88) | 1.31 (0.17–9.96) |
| Fever | 10 (0.5) | 5 (0.9) | 0.48 (0.17–1.40) | 0.28 (0.08–0.92) | 0.35 (0.10–1.25) |
| Constipation | 10 (0.5) | 1 (0.2) | 1.75 (0.29–10.42) | 1.05 (0.16–7.08) | 1.32 (0.17–10.04) |
| Nausea | 9 (0.4) | 4 (0.7) | 0.55 (0.17–1.76) | 0.38 (0.10–1.47) | 0.58 (0.13–2.58) |
| Rash | 9 (0.4) | 2 (0.4) | 1.00 (0.23–4.30) | 1.46 (0.26–8.32) | 1.62 (0.25–10.51) |
| Phlegm accumulation | 8 (0.4) | 1 (0.2) | 1.44 (0.23–8.96) | 0.60 (0.09–4.03) | 0.58 (0.09–3.93) |
| Itching | 7 | 1 | 1.31 | 1.49 | 1.69 |

| | | | | | |
|---|---|---|---|---|---|
| | (0.3) | (0.2) | (0.20–8.42) | (0.17–12.67) | (0.17–16.79) |
| Fatigue | 7 (0.3) | 3 (0.5) | 0.54 (0.14–2.05) | 0.30 (0.07–1.33) | 0.25 (0.06–1.09) |
| Influenza | 5 (0.2) | 3 (0.5) | 0.39 (0.09–1.61) | 3.02 (0.50–18.31) | 4.83 (0.79–29.33) |
| Upper abdominal pain | 5 (0.2) | 2 (0.4) | 0.56 (0.11–2.77) | 1.01 (0.14–7.50) | 0.97 (0.11–8.36) |
| Rhinitis | 5 (0.2) | 4 (0.7) | 0.31 (0.08–1.15) | 0.41 (0.08–2.04) | 0.35 (0.07–1.84) |
| Abdominal discomfort | 4 (0.2) | 1 (0.2) | 0.76 (0.10–5.78) | 1.50 (0.12–18.37) | 0.67 (0.06–7.89) |
| Shortness of breath | 3 (0.1) | 1 (0.2) | 0.58 (0.07–4.94) | 0.41 (0.04–4.72) | 0.53 (0.04–7.46) |
| Headache | 3 (0.1) | 4 (0.7) | 0.20 (0.05–0.89) | 0.33 (0.05–2.17) | 0.33 (0.05–2.41) |
| Anemia | 3 (0.1) | 2 (0.4) | 0.35 (0.06–2.05) | 0.51 (0.06–4.67) | 0.42 (0.04–4.26) |
| Add-on prescriptions | | | | | |
| Expectorants | 62 (3.0) | 21 (3.7) | 0.71 (0.44–1.17) | 0.86 (0.48–1.56) | 1.08 (0.56-2.07) |
| Antipyretics and analgesics | 38 (1.9) | 22 (3.9) | 0.41 (0.24–0.69) | 0.55 (0.29–1.05) | 0.71 (0.35-1.45) |
| Antitussives | 36 (1.8) | 16 (2.8) | 0.55 (0.30–0.99) | 1.17 (0.56–2.47) | 1.51 (0.67-3.38) |
| Antiallergic agents | 35 (1.7) | 13 (2.3) | 0.65 (0.34–1.22) | 1.47 (0.66–3.26) | 1.68 (0.71-3.97) |
| Antiulceratives | 33 (1.6) | 8 (1.4) | 0.95 (0.44–2.04) | 1.06 (0.43–2.61) | 1.27 (0.48-3.36) |
| Antitussives and expectorants | 26 (1.3) | 5 (0.9) | 1.19 (0.47–3.04) | 1.82 (0.60–5.48) | 2.80 (0.85-9.24) |
| Beta-lactam antibiotics | 23 (1.1) | 8 (1.4) | 0.66 (0.30–1.46) | 0.63 (0.25–1.63) | 0.80 (0.29-2.23) |
| Antihypertensives | 23 (1.1) | 3 (0.5) | 1.58 (0.50–4.96) | 1.05 (0.30–3.64) | 0.80 (0.23-2.77) |
| Anti-inflammatory | 15 | 6 | 0.60 | 2.40 | 3.64 |

| | | | | | |
|---|---|---|---|---|---|
| drugs | (0.7) | (1.1) | (0.23–1.53) | (0.73–7.84) | (1.08-12.30) |
| Fluoroquinolones | 13 (0.6) | 5 (0.9) | 0.60 (0.22–1.66) | 0.46 (0.14–1.50) | 0.52 (0.15-1.84) |
| Probiotics | 13 (0.6) | 2 (0.4) | 1.32 (0.33–5.33) | 1.15 (0.24–5.56) | 2.11 (0.37-12.14) |
| Macrolides | 11 (0.5) | 2 (0.4) | 1.10 (0.27–4.53) | 1.39 (0.26–7.35) | 2.04 (0.33-12.66) |
| Magnesium laxatives | 9 (0.4) | 1 (0.2) | 1.54 (0.25–9.40) | 1.46 (0.19–11.08) | 1.09 (0.14-8.48) |
| Steroids | 8 (0.4) | 1 (0.2) | 1.40 (0.22–8.70) | 2.74 (0.32–23.27) | 3.73 (0.38-36.35) |
| Antiemetics | 7 (0.3) | 4 (0.7) | 0.41 (0.12–1.38) | 0.29 (0.07–1.20) | 0.38 (0.08-1.73) |
| Anti-influenza drugs | 6 (0.3) | 1 (0.2) | 1.02 (0.15–6.82) | 6.06 (0.65–56.17) | 10.54 (1.10-100.87) |
| Antivertigo agents | 6 (0.3) | 2 (0.4) | 0.61 (0.13–2.87) | 0.80 (0.12–5.27) | 0.74 (0.10-5.47) |
| Common cold drugs | 3 (0.1) | 2 (0.4) | 0.25 (0.06–2.08) | 2.50 (0.23–27.16) | 2.52 (0.23-27.56) |

Abbreviations: PS, propensity score.

[a] Hazard ratios were estimated using Firth’s penalized likelihood Cox proportional hazards regression to prevent sparse event bias.

[b] The propensity score was calculated using a multivariable logistic regression model incorporating 24 baseline covariates (age, sex, alcohol use, over-the-counter drug use, 11 baseline medical conditions, and 8 concomitant medication classes).

Table S5. Subgroup analysis in patients aged ≥ 65 years: Unadjusted, age- and sex-adjusted, and multivariable-adjusted hazard ratios for acute clinical events and add-on prescriptions associated with molnupiravir versus ensitrelvir use.

| **Events** | **Aged ≥ 65 year** | | **Hazard ratio[a] (95% Confidence Interval)** | | |
|---|---|---|---|---|---|
| | **Molnupiravir (*N* = 1590) *n* (%)** | **Ensitrelvir (*N* = 127) *n* (%)** | **Unadjusted** | **Age and sex sdjusted** | **Multivariate Adjusted[b]** |
| Acute clinical events | | | | | |
| Cough | 51 (3.2) | 4 (3.1) | 0.99 (0.37–2.61) | 1.00 (0.38–2.64) | 0.97 (0.36–2.60) |
| Reduced therapeutic effect | 15 (0.9) | 0 (0.0) | 2.69 (0.15–49.41 | 2.57 (0.14–47.51) | 2.25 (0.11–44.48) |
| Productive cough | 12 (0.8) | 0 (0.0) | 2.16 (0.11–40.99) | 2.16 (0.11–41.18) | 1.99 (0.10–40.85) |
| Oral and pharyngeal pain | 8 (0.5) | 2 (1.6) | 0.29 (0.07–1.26) | 0.32 (0.07–1.44) | 0.39 (0.08–1.92) |
| Nasopharyngitis | 8 (0.5) | 0 (0.0) | 1.46 (0.07–29.96) | 1.56 (0.08–32.33) | 1.62 (0.07–40.01) |
| Diarrhea | 8 (0.5) | 0 (0.0) | 1.42 (0.07–29.21) | 1.60 (0.08–33.27) | 2.02 (0.08–50.72) |
| Vertigo | 10 (0.6) | 0 (0.0) | 1.79 (0.09–35.02) | 2.21 (0.11–43.86) | 2.15 (0.10–45.83) |
| Elevated blood pressure | 7 (0.4) | 1 (0.8) | 0.43 (0.07–2.74) | 0.42 (0.06–2.73) | 0.37 (0.05–2.54) |
| Fever | 8 (0.5) | 2 (1.6) | 0.29 (0.07–1.26) | 0.23 (0.05–1.02) | 0.23 (0.05–1.10) |
| Constipation | 9 (0.6) | 0 (0.0) | 1.63 (0.08–32.54) | 1.56 (0.08–31.66) | 1.72 (0.08–38.75) |
| Nausea | 8 (0.5) | 1 (0.8) | 0.47 (0.08–2.95) | 0.43 (0.07–2.73) | 0.47 (0.07–3.19) |
| Rash | 8 (0.5) | 1 (0.8) | 0.47 (0.08–2.92) | 0.69 (0.11–4.37) | 0.75 (0.11–5.34) |
| Phlegm accumulation | 7 (0.4) | 1 (0.8) | 0.41 (0.06–2.65) | 0.39 (0.06–2.51) | 0.37 (0.06–2.56) |

| | | | | | |
|---|---|---|---|---|---|
| Itching | 6 (0.4) | 1 (0.8) | 0.36 (0.05–2.42) | 0.42 (0.06–2.83) | 0.45 (0.06–3.52) |
| Fatigue | 13 (0.8) | 1 (0.8) | 0.43 (0.07–2.78) | 0.47 (0.07–3.08) | 0.59 (0.08–4.48) |
| Influenza | 0 (0.0) | 0 (0.0) | — | — | — |
| Upper abdominal pain | 3 (0.2) | 1 (0.8) | 0.20 (0.02–1.67) | 0.25 (0.03–2.13) | 0.23 (0.02–2.86) |
| Rhinitis | 3 (0.2) | 1 (0.8) | 0.20 (0.02–1.73) | 0.19 (0.02–1.65) | 0.24 (0.02–2.57) |
| Abdominal discomfort | 3 (0.2) | 0 (0.0) | 0.59 (0.02–17.93) | 0.84 (0.03–27.22) | 0.82 (0.02–29.19) |
| Shortness of breath | 2 (0.1) | 0 (0.0) | 0.43 (0.01–17.58) | 0.36 (0.01–15.14) | 0.26 (0.00–19.78) |
| Headache | 3 (0.2) | 0 (0.0) | 0.60 (0.02–18.42) | 0.78 (0.02–25.09) | 0.97 (0.02–43.709 |
| Anemia | 3 (0.2) | 0 (0.0) | 0.60 (0.02–18.27) | 0.56 (0.02–17.56) | 0.63 (0.02–26.37) |
| Add-on Prescriptions | | | | | |
| Expectorants | 45 (2.8) | 2 (1.6) | 1.49 (0.41–5.39) | 1.48 (0.41–5.36) | 1.41 (0.39–5.18) |
| Antipyretics and analgesics | 23 (1.4) | 5 (3.9) | 0.34 (0.13–0.87) | 0.32 (0.12–0.83) | 0.31 (0.12–0.83) |
| Antitussives | 19 (1.2) | 1 (0.8) | 1.07 (0.19–5.84) | 1.06 (0.19–5.85) | 0.90 (0.16–5.16) |
| Antiallergic agents | 20 (1.3) | 4 (3.1) | 0.37 (0.13–1.05) | 0.43 (0.15–1.23) | 0.46 (0.16–1.33) |
| Antiulceratives | 23 (1.4) | 3 (2.4) | 0.55 (0.17–1.71) | 0.57 (0.18–1.80) | 0.57 (0.17–1.84) |
| Antitussives and expectorants | 19 (1.2) | 0 (0.0) | 3.20 (0.18–57.08) | 3.42 (0.19–61.40) | 3.65 (0.19–71.21) |
| Beta-lactam antibiotics | 20 (1.3) | 1 (0.8) | 1.10 (0.20–6.01) | 1.06 (0.19–5.84) | 1.01 (0.18–5.65) |
| Antihypertensives | 20 (1.3) | 2 (1.6) | 0.66 (0.17–2.51) | 0.65 (0.17–2.52) | 0.60 (0.15–2.38) |
| Anti-inflammatory | 4 (0.3) | 1 (0.8) | 0.25 | 0.23 | 0.23 |

| | | | | | |
|---|---|---|---|---|---|
| drugs | | | (0.03–1.86) | (0.03–1.80) | (0.02–2.18) |
| Fluoroquinolones | 1 (0.7) | 0 (0.0) | 1.88 (0.10–36.23) | 1.73 (0.09–33.51) | 1.58 (0.08–33.25) |
| Probiotics | 9 (0.6) | 0 (0.0) | 1.55 (0.08–30.95) | 1.28 (0.06–25.97) | 1.44 (0.06–32.63) |
| Macrolides | 8 (0.5) | 0 (0.0) | 1.38 (0.07–28.47) | 1.23 (0.06–25.75) | 1.01 (0.04–23.87) |
| Magnesium laxatives | 7 (0.4) | 0 (0.0) | 1.21 (0.06–25.86) | 1.24 (0.06–26.68) | 1.44 (0.06–35.92) |
| Steroids | 5 (0.3) | 0 (0.0) | 0.88 (0.04–20.94) | 1.03 (0.04–24.87) | 1.26 (0.04–39.66) |
| Antiemetics | 6 (0.4) | 1 (0.8) | 0.35 (0.05–2.34) | 0.32 (0.05–2.16) | 0.34 (0.04–2.62) |
| Anti-influenza drugs | 0 (0.0) | 0 (0.0) | — | — | — |
| Antivertigo agents | 4 (0.3) | 0 (0.0) | 0.72 (0.03–18.97) | 0.89 (0.03–24.05) | 1.17 (0.03–43.20) |
| Common cold drugs | 1 (0.1) | 0 (0.0) | 0.26 (0.00–24.15) | 0.26 (0.00–25.30) | 0.35 (0.00–79.62) |

[a] Hazard ratios were estimated using Firth's penalized likelihood Cox proportional hazards regression to handle zero-event categories and prevent sparse event bias. "—" indicates that hazard ratios could not be calculated due to zero events in both treatment groups.

[b] Adjusted for age, sex, alcohol consumption, over-the-counter drug use, hypertension, diabetes mellitus, dyslipidemia, myocardial infarction, heart failure, peptic ulcer, asthma, cancer, hyperuricemia, chronic kidney disease, liver disease, history of hospital admission, concomitant medications (antihypertensives, antidiabetics, lipid-lowering agents, antipsychotics, hypnotics, antiulceratives, and steroids except those for external use), and severe acute respiratory syndrome coronavirus 2 vaccination.
